# Familial 5.29 Mb deletion in chromosome Xq22.1–q22.3 with a normal phenotype: a rare pedigree and literature review

**DOI:** 10.1186/s12920-023-01547-2

**Published:** 2023-05-22

**Authors:** Hui-Hui Xu, Yang Zhang, Zhe-Hang He, Xing-Hong Di, Fei-Yan Pan, Wei-Wu Shi

**Affiliations:** 1 Prenatal Diagnosis Center, Taizhou Hospital, Wenzhou Medical University, Wenzhou, Zhejiang China; 2 Medical Research Center, Taizhou Hospital, Wenzhou Medical University, Wenzhou, Zhejiang China

**Keywords:** Xq22.1–q22.3 deletion, Chromosome aberrations, Birth defect, Copy number variation (CNV), Next-generation sequencing (NGS), Genetic counselling

## Abstract

**Background:**

Xq22.1–q22.3 deletion is a rare chromosome aberration. The purpose of this study was to identify the correlation between the phenotype and genotype of chromosome Xq22.1–q22.3 deletions.

**Methods:**

Chromosome aberrations were identified by copy number variation sequencing (CNV-seq) technology and karyotype analysis. Furthermore, we reviewed patients with Xq22.1–q22.3 deletions or a deletion partially overlapping this region to highlight the rare condition and analyse the genotype–phenotype correlations.

**Results:**

We described a female foetus who is the “proband” of a Chinese pedigree and carries a heterozygous 5.29 Mb deletion (GRCh37: chrX: 100,460,000–105,740,000) in chromosome Xq22.1–q22.3, which may affect 98 genes from *DRP2* to *NAP1L4P2*. This deletion encompasses 7 known morbid genes: *TIMM8A, BTK, GLA, HNRNPH2, GPRASP2, PLP1,* and *SERPINA7*. In addition, the parents have a normal phenotype and are of normal intelligence. The paternal genotype is normal. The mother carries the same deletion in the X chromosome. These results indicate that the foetus inherited this CNV from her mother. Moreover, two more healthy female family members were identified to carry the same CNV deletion through pedigree analysis according to the next-generation sequencing (NGS) results. To our knowledge, this family is the first pedigree to have the largest reported deletion of Xq22.1–q22.3 but to have a normal phenotype with normal intelligence.

**Conclusions:**

Our findings further improve the understanding of the genotype–phenotype correlations of chromosome Xq22.1–q22.3 deletions.This report may provide novel information for prenatal diagnosis and genetic counselling for patients who carry similar chromosome abnormalities.

**Supplementary Information:**

The online version contains supplementary material available at 10.1186/s12920-023-01547-2.

## Background

Birth defects are a major public health problem that lead to miscarriage, foetal death, premature birth and childhood disabilities [1]. In China, approximately 5.6% of newborns are affected by birth defects annually; of these, chromosome aberrations account for more than 80% of the genetic causes, including abnormalities in chromosome number (aneuploidy) or structure, large fragment deletion/duplication, and pathogenic copy number variations (CNVs) [2, 3]. With the implementation of the universal “two child” policy, the proportion of birth defects has increased. This increase might be partly due to the increase in maternal age at delivery, the proportion of mothers with complications, and the number of multiple pregnancies. However, the increase in the number of prenatal screening or prenatal diagnoses for pregnant women of advanced age in China might have alleviated this increasing trend in birth defects [4].

For decades, karyotype analysis has been widely used as the “gold standard” for chromosome aberrations, as it can identify aneuploidy, translocation and inversion of chromosomes. However, karyotyping cannot detect abnormalities in chromosome fragments smaller than 5–10 Mb. Notably, more than 300 types of microdeletion/microduplication syndromes that are caused by CNVs smaller than 5 Mb have been identified, and they account for half of the birth defects caused by chromosome aberrations. CNV sequencing (CNV-seq) technology has brought opportunities and challenges to the detection of chromosome aberrations smaller than 5 Mb. In 2019, genetic experts suggested that CNV-seq could be used as a first-line prenatal diagnosis test for pregnant women who may have foetal chromosome abnormalities in China [5, 6].

Large fragment deletions in chromosome Xq22 might cause neurodevelopmental disorders, including severe intellectual disability and behavioural abnormalities. In this study, we report a female foetus who carries a heterozygous 5.29 Mb deletion in chromosome Xq22.1–q22.3 (including 7 known morbid genes), which was inherited from her healthy mother who had a normal phenotype with normal intelligence.

## Methods

### Karyotype analysis

The pregnant women underwent amniocentesis for karyotype analysis to identify chromosome aberrations of the foetus. In addition, karyotype analysis of peripheral blood were performed in the nonconsanguineous parents to determine the possible causes of chromosome aberration. Using conventional G-banding analysis technology, twenty-five metaphases were analysed at the 550 chromosome band resolution.

### CNV sequencing analysis

CNV sequencing procedures, including DNA extraction, library construction, next-generation sequencing (NGS), bioinformatics analysis, and quality control (QC), were performed in our NGS laboratory with the Ion Torrent platform (BioelectronSeq 4000 sequencing system: Life Technologies, USA) according to the manufacturer’s protocol (Product No. S30030).

## Results

**Fig. 1:**
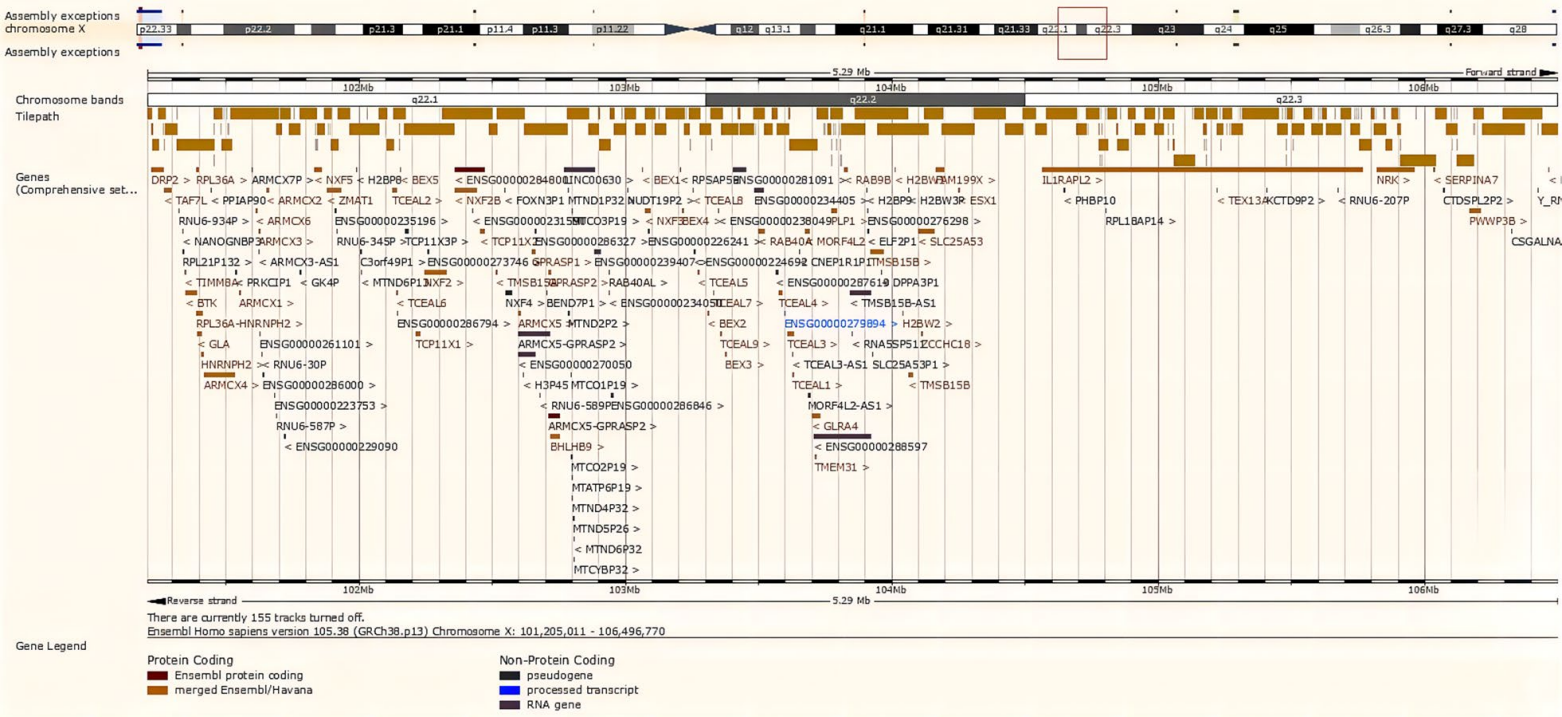
Ensembl genome browser image showing the Xq22.1–q22.3 deletions (GRCh37: ChrX: 100,460,000–105,740,000). Red frame indicate the location of the deletion regions identified in the Chinese pedigree in this study

A healthy pregnant woman, who was 37 years old with G3P1A1, had a 12-year-old healthy daughter. The pregnant woman requested prenatal diagnosis due to advanced maternal age. 3D ultrasound examination showed no evidence of foetal anomalies. She underwent amniocentesis for karyotype analysis and CNV-seq at 23 + 5 weeks gestation at Taizhou Hospital of Zhejiang Province. The foetal karyotype analysis showed a normal female karyotype of 46,XX (Additional file 1). However, the results of CNV-seq analysis indicated a 5.29 Mb deletion in chromosome Xq22.1–q22.3 (GRCh37/hg19: chrX: 100,460,000–105,740,000), which may affect 98 genes from *DRP2* to *NAP1L4P2* according to the Ensembl genome browser (https://asia.ensembl.org/) (Fig. 1) and the ClinGen database (https://www.clinicalgenome.org/) (Additional file 2). According to the DECIPHER database (https://www.deciphergenomics.org), this CNV deletion encompassed 42 OMIM genes, including *DRP2, TAF7L, TIMM8A, BTK, RPL36A, GLA, HNRNPH2, ARMCX1* ~ *6, ZMAT1, BEX1* ~ *5, NXF2* ~ *5, TMSB15A, GPRASP1* ~ *2, BHLHB9, RAB40AL, TCEAL7, RAB40A, TCEAL1, MORF4L2, PLP1, RAB9B, TMSB15B, H2BW1, SLC25A53, ESX1, IL1RAPL2, TEX13A, NRK, and SERPINA7* [7]. Among these OMIM genes, there are 7 known morbid genes, including *TIMM8A, BTK, GLA, HNRNPH2, GPRASP2, PLP1,* and *SERPINA7.* In addition, the nonconsanguineous parents have a normal phenotype and are of normal intelligence. Their intellectual levels have not been precisely tested, but they judged to be normal from their normal social activities.

**Fig. 2:**
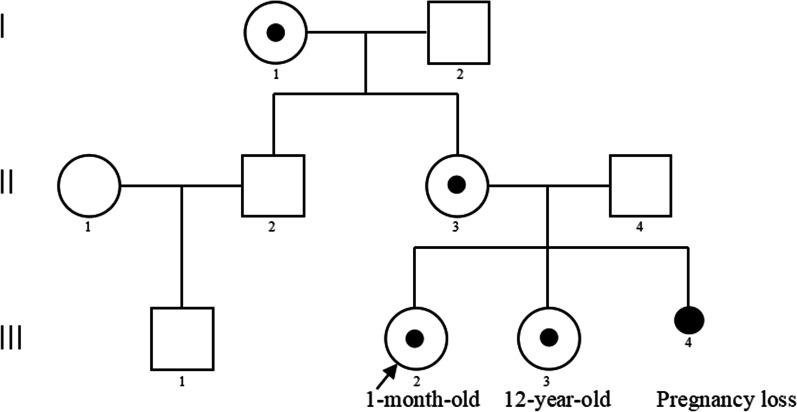
Three-generation pedigree of a Chinese family and carries a heterozygous 5.29 Mb deletion in chromosome Xq22.1–q22.3

**Fig. 3:**
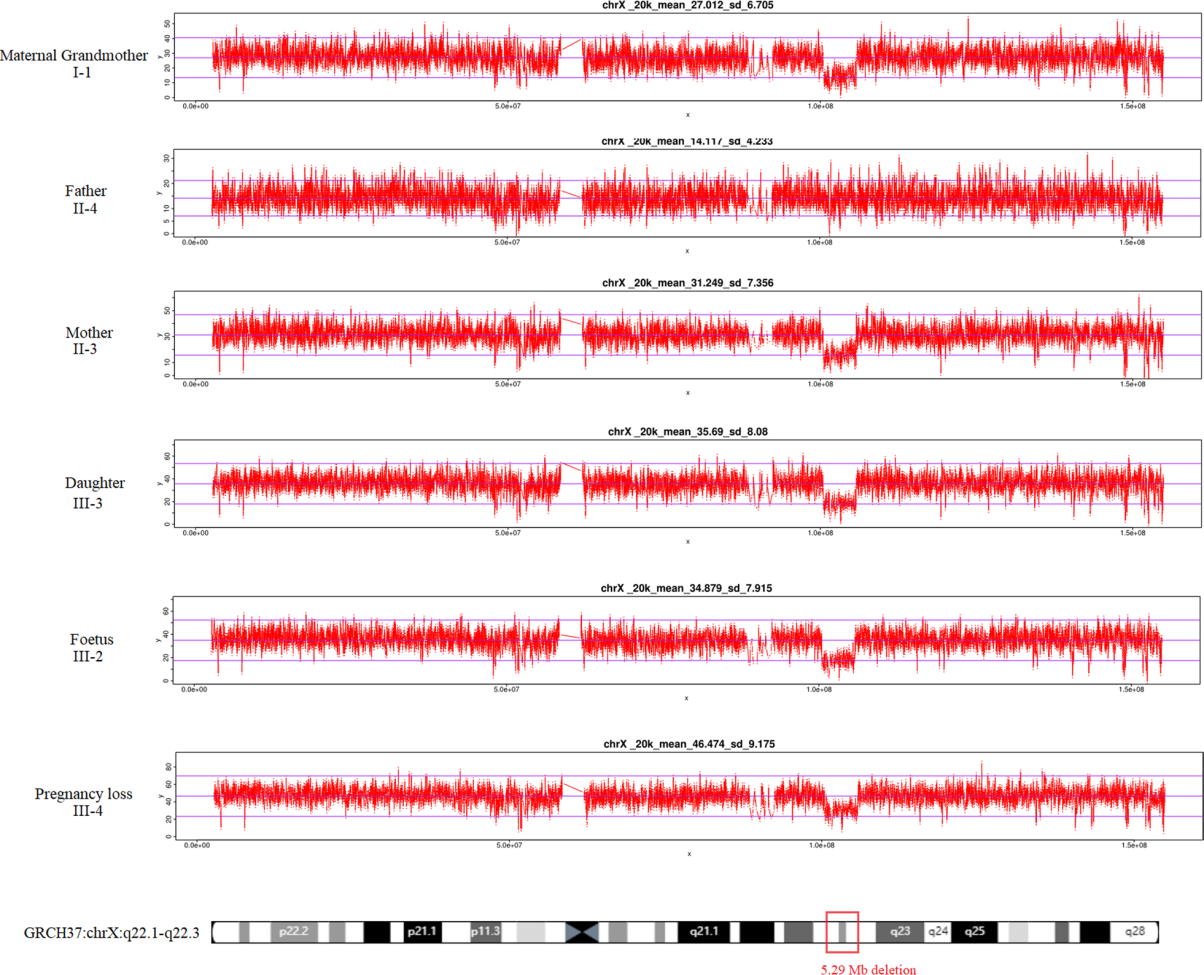
Chromsomal aberrations revealed by CNV-seq analysis are shown with Agilent Genomic Workbench (Agilent Technologies) in chromosome view. X- and Y-axes indicate chromosomal location and signal log2 ratio, respectively. A microdeletion is shown in Xq22.1–q22.3 region (5.29 Mb)

When the pregnant woman had genetic counselling in our prenatal diagnosis centre, we learned that she had a term birth of a healthy girl in 2010 and suffered a termination of pregnancy due to the 46, XXX karyotype of the foetus in 2018. The family wanted to know whether the foetus would have genetic defects after birth. Therefore, we further investigated this pedigree to determine the possible causes of the Xq22.1–q22.3 deletion (Fig. 2). Is it due to parental inheritance or a novel foetal mutation? Further pedigree analysis indicated that the CNV deletion of this foetus was inherited from her healthy mother. Moreover, two more healthy female family members (the pregnant woman’s daughter and mother) were identified to carry the same Xq22.1–q22.3 deletion (Fig. 3). The pregnant woman has a normal clinical phenotype with regular menses and normal fertility. There were no problems during pregnancy or delivery. Her daughter is now 12 years old with normal physical and psychomotor development. Her mother is now 65 years old with normal physical and psychomotor development. Through a genotype–phenotype correlation analysis, although the 5.29 Mb deletion in chromosome Xq22.1–q22.3 was inherited from a normal phenotype parent, it is still considered to be a pathogenic CNV in this pedigree as it contains 7 known morbid genes (*TIMM8A, BTK, GLA, HNRNPH2, GPRASP2, PLP1,* and *SERPINA7*).

After genetic counselling, the couple decided to continue with the pregnancy. On February 28, 2022, a female neonate weighing 4.4 kg and 49 cm in length was born at 39 plus 3 weeks of pregnancy by spontaneous labour. The foetus had a five-minute Apgar score of 10 points, and no abnormal clinical symptoms or signs have been observed to date.

## Discussion

In this rare Chinese pedigree, no abnormality was found in the G-banding karyotype analysis of the foetus or her parents. As the “gold standard” for chromosome aberrations, conventional Giemsa-banding karyotype analysis cannot detect chromosome abnormalities at a resolution of smaller than 5–10 Mb. However, CNV-seq technology provides opportunities and challenges to detect chromosome aberrations smaller than 5 Mb. In this study, CNV-seq analysis of uncultured amniotic fluid cells showed a 5.29 Mb deletion (GRCh37: chrX: 100,460,000–105,740,000) in chromosome Xq22.1–q22.3. It appears that the 5.29 Mb deletion in Xq22.1–q22.3 is a rare chromosome aberration. The foetus inherited this CNV deletion from her healthy mother.

A total of 98 genes were mapped to this 5.29 Mb deletion CNV. This fragment encompasses 7 known morbid genes, *translocase of inner mitochondrial membrane 8A (TIMM8A)*, *Bruton tyrosine kinase (BTK)*, *galactosidase alpha (GLA)*, *heterogeneous nuclear ribonucleoprotein H2 (HNRNPH2)*, *G protein-coupled receptor associated sorting protein 2 (GPRASP2)*, *proteolipid protein 1 (PLP1)*, and *serpin family A member 7 (SERPINA7)*. According to the OMIM database (http://omim.org/), defects in the *TIMM8A* gene are the cause of Mohr–Tranebjaerg syndrome (MTS) [MIM #304700], defects in *BTK* are the cause of X-linked agammaglobulinemia (XLA) [MIM #300755], defects in *GLA* are the cause of Fabry disease (FD) [MIM #301500], defects in *HNRNPH2* are the cause of the bain type of X-linked syndromic intellectual developmental disorder (MRXSB)[MIM #300986], defects in *GPRASP2* are the cause of X-linked deafness-7 (DFNX7) [MIM #301018], defects in *PLP1* are the cause of Pelizaeus–Merzbacher disease (PMD) [MIM #312080] or Spastic paraplegia 2 (SPG2) [MIM #312920], and defects in *SERPINA7* are the cause of Thyroxine-binding globulin quantitative trait locus (TBGQTL) [MIM #300932].

A literature review identified that more than 43 families and 56 cases involving the affected region of Xq22.1–q22.3 deletion or a deletion that partially overlaps have been previously reported [8–31]. None of these previously reported cases had the same CNV deletion as the Chinese pedigree we reported. As shown in Table 1, we analysed the genotype–phenotype correlations of these patients with CNV deletions in chromosome Xq22.1–q22.3. Among them, the phenotype of female cases mainly include severe mental or physical limitations [9, 12–17, 27]. But so far, only one 4-year-old female of Xq22.1 → qter deletion had a normal phenotype [32]. Fortunately, in this Chinese pedigree, all three females with the same Xq22.1–q22.3 deletion have a normal phenotype, most likely due to complete inactivation of the abnormal X chromosomes in females [14, 17]. Notably, no abnormal clinical symptoms or signs have been observed in the fourth female neonate in this Chinese pedigree to date. However, further follow-up will still be necessary to evaluate the phenotype.

**Table 1:** Summary of the genotype–phenotype correlation of chromosome Xq22.1–q22.3 deletions

Author	Age/Sex	Deletion regions^a^ and/or genes	Deletion size	Phenotype
Pelizaeus–Merzbacher disease, PMD /Spastic paraplegia type 2, SPG2 (defects in *PLP1* gene)				
Raskind et al. [8]	35 year/M	Complete deletion of *PLP1* gene	29 kb	Motor development delay, severe dysarthria and scanning speech, gross pendular horizontal nystagmus on lateral gaze to either side, cannot walk, optic disks pale, poorly coordinated dystonic movements of both arms, elbows contractures, both lower extremities spasticity, bilateral ankle clonus, bilateral toes grasping responses and right Bakinski reflex positive, jaw jerk 1 + , snout reflex positive
	17 year/M (His older half-brother)			In the postnatal period: seizure; at age 15 years: alert, little purposeful movement, moving eyebrows and shaking head to answer questions, neck and fingers hyperextended, elbows, wrists, hips, and knees flexion contractures
	4 year/M (His nephew)			Motor developmental delay, spastic diplegia, tendon reflexes hyperactive, bilateral extensor plantar responses, right optic nerve pallor, bilateral increased signal intensity in the periventricular white matter
	59 year/F (His mother)			Knee reflexes hyperactive, upward gaze restrict, smooth-pursuit eye movements coordinated poorly
Inoue et al. [9]	10 year/M	ChrX: 102,993,718–103,510,104	0.5 Mb	Motor development delay, spasticit particularly in lower extremities, dysmyelination, brainstem auditory evoked potentials abnormal
	30 year /F (His mother)			His mother: walking difficulty from third decade. Subsequently, spasticity and personality changes, mental deterioration, cerebral white matter changes
	10 year/M	ChrX: 102,957,289–103,314,254	0.4 Mb	At 18 months: unable to sit unsupported, roll over and no intelligible speech, spasticity; over the next few years: dysarthric; at age 5 years: the cerebrum delayed; at age 7 years: speech dysarthria and slowing, MRI revealed progressive abnormalities in cerebral white matter; at age 10 years: brainstem auditory and somatosensory evoked potentials were abnorma, loss of self-reliance, optic atrophy
	32 year/F (His mother)			bilateral pes cavus deformities of the feet, increased deep tendon reflexes and muscle tone in the lower extremities, perform tandem gait losing
Hübner et al. [10]	unknown	unknown, but complete deletion of *PLP1* and *RAB9L* genes	115 kb	PMD syndrome
	unknown/M (Affected brother)			After birth handicapped
	unknown	unknown, but complete deletion of *PLP1* and *RAB9L* genes	115 kb	PMD syndrome
	unknown/M (Affected brother)			After birth handicapped
Lee et al. [11]	unknown/M	ChrX: 103,009,829–103,214,881	190 kb	PMD syndrome
Torisu et al. [12]	2 year/M	ChrX: 103,018,951–103,092,038	73 kb	Spastic quadriplegia, mental retardation, microcephaly, brainstem auditory evoked potentials prolonged, hypomyelination, axonal involvement, nerve conduction velocity of the lower extremities decreased
Matsufuji et al. [13]	29 year/M	ChrX: 103,033,333–103,066,899 (Partial deletion of *PLP1* gene)	33 kb	Spastic quadriplegia, dysarthria, ataxia, dysphagia, intellectual delay
	59 year/F (His mother)			His mother has spastic diplegia, dementia
	31 year/F (His sister)			His sister has spastic diplegia, motor developmental delay, dysphagia from childhood
Yamamoto et al. [14]	6 year/F	ChrX: 101,365,862–105,847,036	4.4 Mb	Wide intermamillary distance, constipation, low-set ears, anterior hypopituitarism, large for gestational age, tall stature, blepharophimosis, high palate, narrow palate, prominent nasal bridge, wide nasal bridge, broad toe, hypotonia, joint laxity, macrocephaly, micrognathia, overlapping toe, short foot, abnormal CNS myelination, hydrocephalus, hypoplasia of the corpus callosum
	3 year/F	ChrX: 100,659,116–105,523,589	4.8 Mb	Developmental delay, hypersomnia, white matter hypoplasia, myelination delay, corpus callosum hypoplasia, ventriculomegaly. language skills lose, cannot sit or walk, triangular face, strabismus, jaw prominent, pesequinovarus, intellectual disability
	16 year/F	ChrX: 100,907,884–103,982,269	3 Mb	Scoliosis, bilateral hearing loss, constipation, advanced bone age, aphasic, incontinent, hair growing slow, bifrontal narrowing, deep-set eyes, a prominent nasal bridge, full upper lip, a prominent jaw, deep palmer creases and prominent volar pads
	1 year/F	ChrX: 101,982,865–102,233,526	0.25 Mb	Motor developmental delay, bilateral sensorineuronal deafness
	7 year/F	ChrX: 102,959,459–103,044,544 (Partial deletion of *PLP1* gene)	85 kb	Early infancy: psychomotor developmental delay; at 18 months: cannot sit; at age 6 years: cannot walk, delayed myelination, aphasic, incontinent, pain perception impaired, sleeps poorly, strabismus, intellectual disability, leukodystrophy
Brender et al. [15]	16 year/F	Deletion of *NGFRAP1, TCEAL1, MORFL2, PLP1, RAB9B,* and *H2BFWT* genes	712 kb	At birth: nystagmus; at 6 months: delayed motor development, spasticity; at age 3 years: aphasia; at 14 year: loss of expression, nystagmus, exotropia, agitation; at 7 years: cannot walking; at ages of 4, 9, 13, and 17: the frontal horn to the occipital horn of the lateral ventricles bilaterally linear increased; at 16 years: onset seizure disorder, Electroencephalogram demonstrated abundant bursts of generalized spike, polyspike, and slow wave activity; at 17 years: remained nonverbal
Kinoshita et al. [16]	3 year/F	Del(X)(q22.1q22.2) / Deletion of 39 genes, including *PLP1* gene	2.26 Mb	Incomplete lung formation, feeding difficulty, hydration, milestones delayed, communicate, recognize and identify lose, cannot walk, emotions lose, broad forehead, small pointed nose, left eye strabismus, thin upper lip, dental decay, grasping difficult, arms and legs strength strength
Hijazi et al. [17]	13 year/F	ChrX: 100,866,604–103,411,980	2.5 Mb	Strabismus, posterior white matter signal, hypotonia followed by spasticity, gastroesophageal reflux disease, poor weight gain, constipation
	9 year/F	ChrX: 102,615,641–103,309,503	693 kb	Strabismus, nystagmus, delayed myelination, thin corpus callosum, cerebral atrophy, hypotonia mixed with spasticity, gastroesophageal reflux disease, facial dysmorphic features
	3.5 year/F	ChrX: 101029649-106702784	5.6 Mb	Strabismus, delayed myelination in parietal/periventricular regions, hypotonia followed by dystonia, gastroesophageal reflux disease, abnormal brain auditory evoked potential, dysmorphic features
	8 year/F	ChrX: 102,066,350–105,409,822	3.3 Mb	Strabismus, left amblyopia, diffuse hypomyelination, partially progressed myelination, thincorpus callosum, white matter atrophy, hypotonia then spasticity, gastroesophageal reflux disease, constipation, Seizure, ventricular septal defect, decreased bone mineral density, hypothyroidism
	unknown/F	ChrX: 102,436,725–105,520,605	3.0 Mb	Unknown
	15 year/M	ChrX: 103,029,773–103,036,548	6.7 kb	Strabismus, diffuse hypomyelination, thin corpus callosum, brain atrophy, spasticity, dystonia, Seizure, extended latency in brain auditory evoked potential, peripheral neuropathy
	16 year/M	ChrX: 102,967,297–103,038,606	71 kb	Strabismus, periventricular white matter change, hypotonia, spasticity, gastroesophageal reflux disease, poor weight gain, abnormal electroencephalogram, initially diagnosed as cerebral palsy with Periventricular leukomalacia
	unknown/M	ChrX: 102,543,473–103,398,234	854 kb	Unknown
X-linked agammaglobulinaemia, XLA/Mohr–Tranebjaerg syndrome, MTS (defects in *BTK/DDP1/TIMM8A* gene)^b^				
Jin et al. [18]	9 year/M	Partial deletion of *BTK* gene andcomplete deletion of *deafness/ dystonia peptide* (*DDP*) gene	21 kb	At 12 months: deafness, dystonia, mental deficiency, recurrent infections; at age 2 years: sensorineural deafness; at age 5 years: dystonia
Richter et al. [19]	6 year/M	Partial deletion of *BTK* gene andcomplete deletion of *DDP* gene	19 kb	At 10 months: pseudomonas aeruginosa sepsis, severe oral aphthous, cutaneousnecrotic lesions, leukopenia andneutropenia, XLA; at age 3–4 years: language skills not developing
	9 year/M	Partial deletion of *BTK* gene andcomplete deletion of *DDP* gene	4.2 kb	At 8 months: profound neutropenia, low levels of serum immunoglobulins, XLA; at age 3–4 years: speech definitely stop developing
	14 year/M	Partial deletion of *BTK* gene andcomplete deletion of *DDP* gene	7 kb	At 18 months: otitis, conjunctivitis, upper and lower respiratory tract infections; at 30 months: P. aeruginosa sepsis, necrotic skin lesions, neutropenia, profound hypogammaglobulinemia, B cells absence, XLA, speech delayed, hearing loss, emotional instability, attention deficit disorder, learning disabilities, auditory and visual processing defects
Pizzuti et al. [20]	24 year/M	Partial deletion of *BTK* gene andcomplete deletion of *DDP* gene	unknown	At age 2 years: bilateral hearing loss, recurrent infections, bruton agammaglobulinemia; at age 15 years: visual lose; at age 19 years: writing difficulties, visual acuity, hearing impaired, the right upper limb dystonic posturing, intellectual deficit
Sedivá et al. [21]	33 year/M	Deletion includes the last exon of the *BTK* gene and both exons of the *TIMM8A* gene	30 kb	Deafness, abnormal speech, aggressive behavior, muscle wasting
	25 year/M (Brother of 33 year old man)			Respiratory infections, progressive deafness
	6 year/M	Deletion includes the exons 6–19 of the *BTK* gene and both exons of the *TIMM8A* gene	22 kb	At 3 months: respiratory infections; at 7 months, XLA, psychomotor retardation, speech impairment; at age 4 years: sensorineural hearing loss
	5 year/ M (Brother of 6 year oldboy)			At 2 months: XLA; at 7 months: acute bronchopneumonia; since 16 months: chronic bronchitis; at age 2.5 years: speech development delayed
	13 year/M	Deletion of *BTK, TIMM8A, TAF7L,* and *DRP2* genes	196 kb	At 6 months: respiratory distress, pneumonia, neutropenia; at 8 months: XLA, pneumonia; at age 3 years: language and motor development delay, hearing loss
	6 year/M (died)	Deletion includes the last exon of the *BTK* gene and the entire *TIMM8A* gene	20 kb	At age 6 years: progressive dystonia, neurological impairment, general wasting, died
Jyonouchi et al. [22]	6 year/M	ChrX: 100,288,859–100,453,630	155 kb	At 5–6 months: bacterial pneumonia, agammaglobulinemia; at age 2–3 years: speech delay, furuncles on trunk and extremities, dental caries, malnourished, hearing loss, speech delay, sinopulmonary infection, circulating CD19 + B cells absence, IgA, IgM, and IgE undetectable
	6 year/M (Two identical twins)			
Brookes et al. [23]	28 year/M	Deletion included exons 17–19 of *BTK* and exon 1 of *DDP1/TIMM8a* genes	6 kb	At 4 months: acute otitis media; at age 1 years: otitis media, recurrent sinusitis, viral upper respiratory tract infections, cutaneous staphylococcus infection, neutropenia, panhypoglobulinemia; at age 5 years: XLA, B lymphocytes lack, communication limited, receptive and expressive language delay, sensorineural hearing loss, speech and language delay persisted
Arai et al. [24]	15 year/M	Deletion included exons 16–19 of *BTK* and *TIMM8a* genes	63 kb	At age 1 years: deafness; at age 7 years: XLA
	10 year/M	Deletion included exons 6–19 of *BTK* and *TIMM8a, TAF7L, DRP2* genes	149.7 kb	At 12 months: otitis media, sinusitis; at 18 months: deafness, autism; at age 8 years: agammaglobulinemia, lack of circulating B cells, XLA, hearing losses
Shaker et al. [25]	27 year/M	Deletion of *BTK, TIMM8A*, and *TAF7L* genes	111 kb	At 11 months: XLA, acute-onset bilateral flaccid paralysis, lower extremities sensory loss, loss of reflexes, pain, and temperature below the T10 level, low levels of immunoglobulins, B-cells absent, spastic lower extremity paraplegia; at age 2 years: hearing loss; at age 20 years: metastatic testicular seminoma
Szaflarska et al. [26]	6 months/M	ChrX: 100,601,727–100,617,576	16 kb	At 5 months: skin abscesses; at 6 months: generalised purulent skin infection, fever and eutropenia; at 7 months: pneumonia, low concentrations of serum immunoglobulins, absolute number of T cells elevated, B lymphocytes absent
Other syndromes				
Grillo et al. [27]	7 year/F	ChrX: 100,934,364–102,047,069	1.1 Mb	At birth: asphyxia, cleft palate surgically; at 18 months: deambulation and speech absent; at age 3 years: sleep tremors; at age 4 years: microbrachycephaly, muscle hypotonia, an unspecific periventricular white matter alteration at cerebral; at age 7 years: mental retardation, hypertricosis on upper limbs, distal muscle hypotrophy of lower limbs, scoliosis and facial ysmorphisms suchascoarse face, small forehead, thick lips, smooth philtrum and low set ears, autistic spectrum disorder, stereotypic movements, self-mutilation
	42 year/F (His mother)		1.1 Mb	Mental retardation, short stature, brachycephaly, epilepsy, a borderline personality disorder
Shimojima et al. [28]	12 year/M	ChrX: 105,167,104–106,028,458	862 kb	At birth: overweight, scant scalp hair, forehead prominent, cleft lip and palate, psychomotor development, hypotonia, episodes of febrile seizures; at age 4 years: scoliosis, orthostatic hypotension; at 6 years: right eye cataract, retinal detachment, highly arched eyebrows, epicanthus, left internal strabismus, flat nose, post-operative cleft lip, thin extremities, generalized hypotonia, extremities hyporeflexia, joints hypermobility. generalized skin hyperextensibility, hair growth slow, speak loss
Labonne et al. [29]	11 year/F	ChrX: 102,882,657-102,987,229	105 kb	At 8 weeks: manifesting nystagmus; at 8 months: cannot sitting or weight bearing; at 9 months: gross motor skills delayed; at 15 months: develop movement patterns, slow; at 20 months: developmental delay, cannot crawling; at 2 years and 1 month: generalized tonic clonic seizure, fever; at 2 years and 3 months: cannot loading; at 2 years and 8 months: development delayed, walked with a wide spaced ataxic gait, hand movements displayed, stare and look blankly; at 3 years and 4 months: deep blue, lightly pigmented irides, displayed frontal bossing, a flat occiput, prominent chin, fifth finger clinodactyly; at 4 years and 1 month: sleep patterns disturbed; at 4 years and 7 months: global developmental delay, hypermetropic astigmatism, minor jerky eye movement; at 5 years: learning difficulties, intermittent episodes of distress, glycosuria, stereotypic movements, walk in a side-to-side stepping, poor coordination, cannot speech, heart murmur, constipation, eating nonfood, cannot independent; at age 6 years: chicken pox; at age 7 years: abdominal discomfort
Cao et al. [30]	8 week/M	ChrX: 100,857,290–101,991,488	1.1 Mb	Thrive failure, hypoglycemia, subtle dysmorphic features, umbilical hernia, hypotonia with spasticity in the lower extremities, focal enlargement of frontal temporal lobe, respiratory failure with thoracic insufficiency syndrome, tracheomalacia, and laryngomalacia
Shirai et al. [31]	17 month/ M	ChrX: 101,381,936–102,754,792	1.4 Mb	After birth: respiratory failure, suspected laryngomalacia and laryngeal wheezing, dysphagia, congenital nasolacrimal duct cyst; at 9 months: tonic seizures, distinctive facial features, including mid-face hypoplasia, micrognathia, redundant nuchal skin, extremities hypertonus, cerebral volume reduced, cannot control head, roll over or make eye contact lose, psychomotor developmental delay, bedridden
Normal phenotype with Xq22.1–q22.3 deletions				
Vaglio et al. [32]	4 year/F	Deletion of a terminal Xq spanning Xq22.1→qter	unknown	Normal
This study	1 month/F	ChrX: 100,460,000–105,740,000	5.28 Mb	Normal
	12 year/F			Normal
	37 year/F			Normal
	65 year/F			Normal

^a^Genomic positions referred to build19

^b^In the same chromosomal region, located 770 bp centromerically of the *BTK* gene, is the gene originally named *DDP1*. The HUGO Gene Nomenclature Committee has named this gene *TIMM8A*

In addition, we focus on the genetic patterns of these morbid genes. Mohr–Tranebjaerg syndrome is caused by mutations in the *TIMM8A* gene, which is a rare X-linked recessive disorder resulting in early-onset hearing impairment, progressive visual deterioration, and gradual dystonia. Some female carriers showed signs of minor neuropathy and mild hearing impairment [33, 34]. Fabry disease is a rare X-linked lipid storage disorder caused by a deficiency or absence of lysosomal alphagalactosidase A, which encoded by *GLA* gene. It is worth noting that heterozygous women should not be called carriers because they often been reported with a wide range of clinical symptoms. The early clinical manifestations mainly include acroparesthesias, angiokeratomas, pain crisis, and cornea verticillata, among other abnormalities. It therefore appears that Fabry disease affects both hemizygotes and heterozyotes, and should be considered an X-linked dominant disorder [35, 36]. Pelizaeus–Merzbacher disease is an X-linked recessive central nervous system disorder, which belongs to the group of hypomyelinating leukodystrophy (HLD1). PMD principally affect males and occasionally observed in carrier females, which is characterized clinically by nystagmus, spastic quadriplegia, ataxia, and developmental delay [9, 17, 37]. In addition, there was no report of male patients with large fragment Xq22 deletions. This is probably because larger Xq22 deletions may lead to embryonic lethality in males, since male patients with smaller nullisomy in the vicinity show more severe developmental delay [8, 10–12, 17].

## Conclusions

X chromosomal deletions are infrequent findings in prenatal diagnosis and present a difficult counselling challenge when they occur. Genotype–phenotype correlation analysis can provide reliable clinical genetic counselling for chromosome abnormality reports. In addition, the X-inactivation pattern could provide an opportunity for more informative genetic counselling when a de novo CNV deletion in the X chromosome is detected.

## Supplementary Information

**Additional file 1**. G-banded karyotypes of the foetus and her parent.

**Additional file 2**. ClinGen database shown 5.29 Mb deletion in chromosome Xq22.1–q22.3 affect 98 genes from *DRP2* to *NAP1L4P2*.

## Data Availability

All data generated during this study are included in this published article.

## Abbreviations

*BTK*: Bruton tyrosine kinase
CNV: Copy number variation
DFNX7: X-linked deafness-7
FD: Fabry disease
*GLA*: Galactosidase alpha
*GPRASP2*: G protein-coupled receptor associated sorting protein 2
*HNRNPH2*: Heterogeneous nuclear ribonucleoprotein H2
MRXSB: X-linked syndromic intellectual developmental disorder
MTS: Mohr–Tranebjaerg syndrome
NGS: Next-generation sequencing
OMIM: Online Mendelian Inheritance in Man
*PLP1*: Proteolipid protein 1
PMD: Pelizaeus–Merzbacher disease
*SERPINA7*: Serpin family A member 7
SPG2: Spastic paraplegia 2
TBGQTL: Thyroxine-binding globulin quantitative trait locus
*TIMM8A*: Translocase of inner mitochondrial membrane 8A
XLA: X-linked agammaglobulinemia
