# Supplementary figures and images for: Familial 5.29 Mb deletion in chromosome Xq22.1–q22.3 with a normal phenotype: a rare pedigree and literature review
Source: BMC Med Genomics. 2023 May 22;16:111. doi: 10.1186/s12920-023-01547-2 (PMC10201758; doi:10.1186/s12920-023-01547-2)

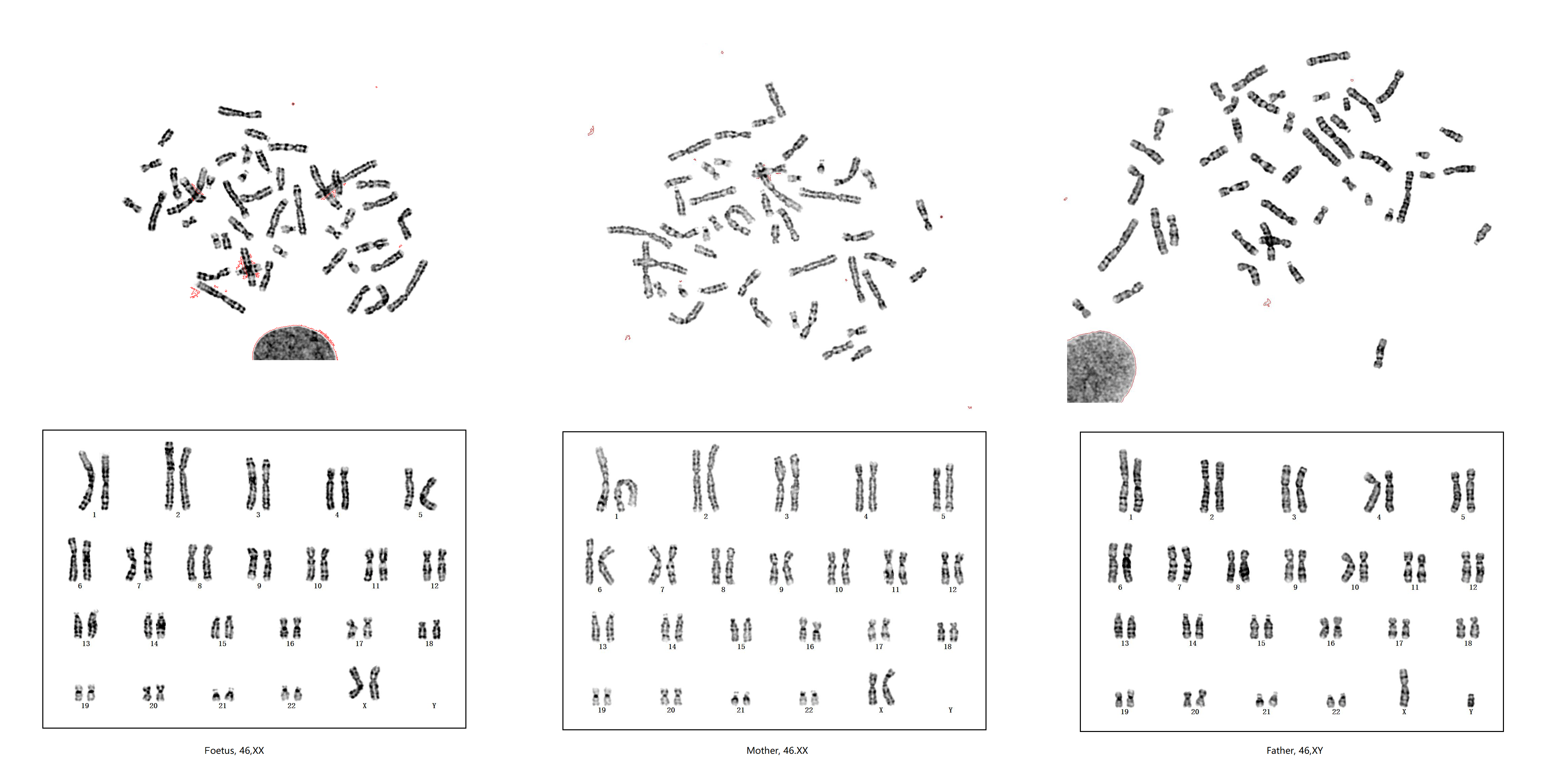

Supplement: Supplementary file 1 — Additional file 1. G-banded karyotypes of the foetus and her parent. [file 12920_2023_1547_MOESM1_ESM.tif]
