# Supplementary material for: Familial 5.29 Mb deletion in chromosome Xq22.1–q22.3 with a normal phenotype: a rare pedigree and literature review
Source: BMC Med Genomics. 2023 May 22;16:111. doi: 10.1186/s12920-023-01547-2 (PMC10201758; doi:10.1186/s12920-023-01547-2)
Supplement: Supplementary file 2 — Additional file 2. ClinGen database shown 5.29 Mb deletion in chromosome Xq22.1–q22.3 affect 98 genes from DRP2 to NAP1L4P2. [file 12920_2023_1547_MOESM2_ESM.pdf]

Gene	Cytoband	Chromosome	Start	Stop	Relationship	Curations	Last Eval.
DRP2	Xq22.1	23	100474775	100519491	Contained		
TAF7L	Xq22.1	23	100523245	100548071	Contained		
RNU6-934P	Xq22.1	23	100580392	100580498	Contained		
NANOGNBP3	Xq22.1	23	100592300	100593195	Contained		
RPL21P132	Xq22.1	23	100595577	100595963	Contained		
TIMM8A	Xq22.1	23	100600649	100603730	Contained		03/24/2021
BTK	Xq22.1	23	100604438	100645784	Contained		03/24/2021
RPL36A	Xq22.1	23	100645999	100651143	Contained		
RPL36A-HNRNP...	Xq22.1	23	100645999	100669121	Contained		
GLA	Xq22.1	23	100652791	100662913	Contained		03/24/2021
HNRNPH2	Xq22.1	23	100663210	100669121	Contained		
ARMCX4	Xq22.1	23	100673266	100790975	Contained		
ARMCX1	Xq22.1	23	100805530	100809683	Contained		
ARMCX7P	Xq22.1	23	100852349	100853046	Contained		
ARMCX6	Xq22.1	23	100870115	100872990	Contained		
ARMCX3	Xq22.1	23	100878141	100882833	Contained		
RNU6-30P	Xq22.1	23	100888995	100889100	Contained		
ARMCX2	Xq22.1	23	100910267	100914835	Contained		
RNU6-587P	Xq22.1	23	100946553	100946658	Contained		
GK4P	Xq22.1	23	101033439	101035626	Contained		
NXF5	Xq22.1	23	101087085	101112549	Contained		05/17/2012
ZMAT1	Xq22.1	23	101137262	101187000	Contained		
RNU6-345P	Xq22.1	23	101170562	101170668	Contained		
MTND6P13	Xq22.1	23	101263497	101264019	Contained		
TCEAL2	Xq22.1	23	101380651	101382684	Contained		
TCEAL6	Xq22.1	23	101393474	101397453	Contained		
BEX5	Xq22.1	23	101408684	101410949	Contained		
TCP11X3P	Xq22.1	23	101428072	101439994	Contained		
TCP11X1	Xq22.1	23	101470280	101481789	Contained		
NXF2	Xq22.1	23	101502161	101581636	Contained		
NXF2B	Xq22.1	23	101615316	101694929	Contained		
TCP11X2	Xq22.1	23	101715240	101726732	Contained		
TMSB15A	Xq22.1	23	101768610	101771667	Contained		
FOXN3P1	Xq22.1	23	101801667	101803142	Contained		
NXF4	Xq22.1	23	101804893	101826621	Contained		
ARMCX5	Xq22.1	23	101854134	101859087	Contained		
ARMCX5-GPRA...	Xq22.1	23	101854276	102162992	Contained		
GPRASP1	Xq22.1	23	101906411	101914011	Contained		
RNU6-589P	Xq22.1	23	101933636	101933741	Contained		
BEND7P1	Xq22.1	23	101960706	101961294	Contained		
GPRASP2	Xq22.1	23	101967376	101972661	Contained		
BHLHB9	Xq22.1	23	101975671	102008468	Contained		
LINC00630	Xq22.1	23	102024081	102219451	Contained		
MTND1P32	Xq22.1	23	102040606	102041532	Contained		
MTND2P2	Xq22.1	23	102041742	102043316	Contained		
MTCO1P19	Xq22.1	23	102051079	102052121	Contained		
MTCO2P19	Xq22.1	23	102052459	102053056	Contained		
MTATP6P19	Xq22.1	23	102053324	102054488	Contained		
MTCO3P19	Xq22.1	23	102054391	102054911	Contained		
MTND4P32	Xq22.1	23	102054894	102056019	Contained		
MTND5P26	Xq22.1	23	102059560	102061048	Contained		
MTND6P32	Xq22.1	23	102061092	102061574	Contained		

Gene	Cytoband	Chromosome	Start	Stop	Relationship	Curations	Last Eval.
MTCYBP32	Xq22.1	23	102061669	102062752	Contained		
RAB40AL	Xq22.1	23	102192200	102193228	Contained		
NUDT19P2	Xq22.1	23	102261999	102262992	Contained		
BEX1	Xq22.1	23	102317579	102319099	Contained		
NXF3	Xq22.1	23	102330738	102348071	Contained		
RPSAP59	Xq22.1	23	102460937	102461827	Contained		
BEX4	Xq22.1	23	102470020	102472174	Contained		
TCEAL8	Xq22.1	23	102507923	102510083	Contained		
TCEAL5	Xq22.1	23	102528619	102531678	Contained		
BEX2	Xq22.2	23	102564274	102565918	Contained		
TCEAL7	Xq22.2	23	102585167	102587254	Contained		
TCEAL9	Xq22.2	23	102611434	102613390	Contained		
BEX3	Xq22.2	23	102631251	102633092	Contained		
RAB40A	Xq22.2	23	102754058	102774417	Contained		
LINC02589	Xq22.2	23	102785695	102809881	Contained		
TCEAL4	Xq22.2	23	102831159	102842657	Contained		
TCEAL3	Xq22.2	23	102862891	102864855	Contained		
TCEAL1	Xq22.2	23	102883644	102885881	Contained		
MORF4L2	Xq22.2	23	102930426	102942975	Contained		
MORF4L2-AS1	Xq22.2	23	102942212	102947488	Contained		
GLRA4	Xq22.2	23	102962272	102983552	Contained		
TMEM31	Xq22.2	23	102965837	102968960	Contained		
RAB9B	Xq22.2	23	103031252	103087187	Contained		
PLP1	Xq22.2	23	103031434	103047548	Contained		04/12/2021
RNA5SP511	Xq22.2	23	103105726	103105840	Contained		
TMSB15B-AS1	Xq22.2	23	103136651	103174131	Contained		
ELF2P1	Xq22.2	23	103163345	103166286	Contained		
TMSB15B	Xq22.2	23	103173738	103229008	Contained		
DPPA3P1	Xq22.2	23	103215901	103216962	Contained		
H2BW4P	Xq22.2	23	103230502	103232935	Contained		
H2BW1	Xq22.2	23	103265719	103268259	Contained		
H2BW2	Xq22.2	23	103294523	103297021	Contained		
SLC25A53	Xq22.2	23	103343898	103401690	Contained		
ZCCHC18	Xq22.2	23	103357214	103360533	Contained		
FAM199X	Xq22.2	23	103411134	103440583	Contained		
ESX1	Xq22.2	23	103494719	103499614	Contained		
IL1RAPL2	Xq22.3	23	103810880	105011822	Contained		
PHBP10	Xq22.3	23	103891726	103892751	Contained		
RPL18AP14	Xq22.3	23	104048549	104049177	Contained		
TEX13A	Xq22.3	23	104463611	104465378	Contained		
KCTD9P2	Xq22.3	23	104650184	104653553	Contained		
RNU6-207P	Xq22.3	23	104920065	104920170	Contained		
NRK	Xq22.3	23	105066532	105202602	Contained		
SERPINA7	Xq22.3	23	105276426	105282718	Contained		
PWWP3B	Xq22.3	23	105412298	105452949	Contained		
NAP1L4P2	Xq22.3	23	105710010	105710596	Contained		
